# P-1206. Association of Sex Assigned at Birth and Sexual Orientation with Antimicrobial Susceptibility of Baseline Neisseria gonorrhoeae Isolates from Participants Recruited in the Global Zoliflodacin Phase 3 Randomized Controlled Trial

**DOI:** 10.1093/ofid/ofaf695.1399

**Published:** 2026-01-11

**Authors:** Sarah McLeod, Esther Bettiol, Varalakshmi Elango, Drew Lewis, Alison Luckey

**Affiliations:** Innoviva Specialty Therapeutics, Inc., Waltham, MA; Global Antibiotic R&D Partnership, Geneva, Geneve, Switzerland; Global Antibiotic R&D Partnership, Geneva, Geneve, Switzerland; Innoviva Specialty Therapeutics, Waltham, Massachusetts; Global Antibiotic R&D Partnership (GARDP), Geneva, Geneve, Switzerland

## Abstract

**Background:**

Zoliflodacin is an oral first-in-class spiropyrimidinetrione bacterial topoisomerase inhibitor with potent *in vitro* activity against multidrug-resistant strains of *Neisseria gonorrhoeae*. The efficacy and safety of a single oral 3 g dose of zoliflodacin in treating uncomplicated gonorrhea were evaluated in a global Phase 3 randomized controlled trial, demonstrating non-inferiority to the standard-of-care treatment (a single intramuscular 500 mg dose of ceftriaxone plus a single oral 1 g dose of azithromycin). The trial showed similar efficacy rates across participant subgroups defined by sex assigned at birth and sexual orientation. Antibiotic susceptibilities of *N. gonorrhoeae* baseline isolates from subgroups of participants based on sex assigned at birth and sexual orientation were analyzed by anatomical site of infection and treatment arm.

**Figure:**
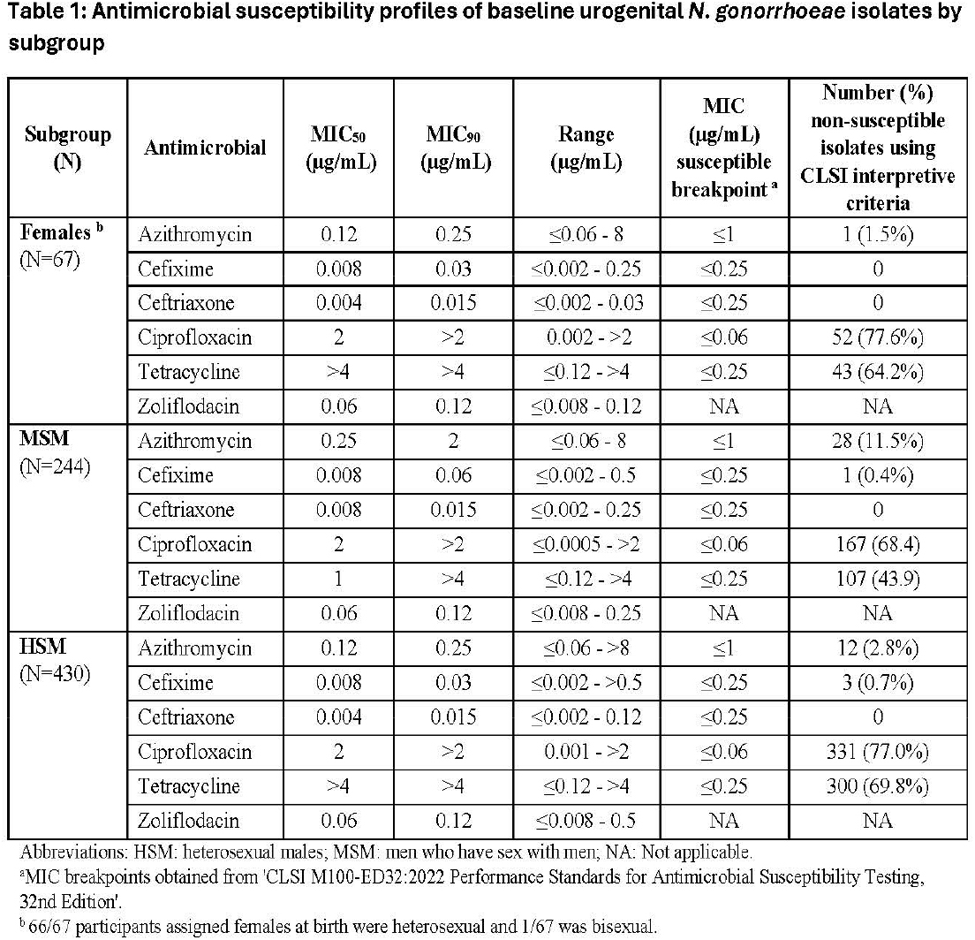

**Methods:**

Antibiotic susceptibility profiles of 936 baseline *N. gonorrhoeae* isolates from trial participants were determined using agar dilution MICs following CLSI guidelines for azithromycin, cefixime, ceftriaxone, ciprofloxacin, tetracycline and zoliflodacin. Isolates were obtained from assigned females (F) and males (M) at birth from urogenital (n= 67 F; 674 M), pharyngeal (n =13 F; 68 M) and rectal (n =22 F; 92 M) sites of infection.

**Results:**

Table 1 shows that the azithromycin MIC_90_ was 0.25 µg/mL for urogenital isolates from females and heterosexual males (HSM) and 2 µg/mL for those from men who have sex with men (MSM), with 1.5%, 2.8% and 11.5% of isolates resistant to azithromycin in each group, respectively. Similar results were observed for pharyngeal and rectal isolates. Four isolates (1 pharyngeal, 1 urethral and 2 rectal) with ceftriaxone MICS of ≥ 0.25 µg/mL were isolated from MSM. Resistance rates to ciprofloxacin and tetracycline were high across all subgroups. Zoliflodacin MICs were within the wildtype distribution and were similar between subgroups at all anatomical sites. MICs of all tested antibiotics were comparable between trial treatment arms.

**Conclusion:**

The largest difference in susceptibility profiles was observed for azithromycin across all anatomical sites, with higher MIC_50_, MIC_90_ and resistance rate in MSM compared to females and HSM. Zoliflodacin MICs remained consistent across all subgroups.

**Disclosures:**

Sarah McLeod, PhD, Innoviva Specialty Therpeutics: Employee|Innoviva Specialty Therpeutics: Stocks/Bonds (Public Company) Drew Lewis, MD, MTM&H, FACP, Innoviva Specialty Therapeutics: Stocks/Bonds (Public Company)

